# Expanding long-acting contraceptive options: a prospective cohort study of the hormonal intrauterine device, copper intrauterine device, and implants in Nigeria and Zambia

**DOI:** 10.1016/S2214-109X(21)00318-1

**Published:** 2021-08-30

**Authors:** Aurélie Brunie, Kayla Stankevitz, Anthony Adindu Nwala, Masauso Nqumayo, Mario Chen, Kendal Danna, Kayode Afolabi, Kate H Rademacher

**Affiliations:** a Global Health, Population and Nutrition, FHI 360, Washington, DC, USA; b Global Health, Population and Nutrition, FHI 360, Durham, NC, USA; c Reproductive Health and Family Planning, Society for Family Health, Abuja, Nigeria; d Research Monitoring and Evaluation Department, Society for Family Health, Lusaka, Zambia; e Sexual and Reproductive Health Department, Population Services International, Washington, DC, USA; f Reproductive Health Division, Federal Ministry of Health, Abuja, Nigeria

## Abstract

**Background:**

30 years after the introduction of the levonorgestrel-releasing intrauterine device in Europe, several sub-Saharan African countries are seeking to broaden access to this contraceptive method. In this study, we aimed to assess 12-month continuation of the hormonal intrauterine device, copper intrauterine device, and implants, as well as to assess women's experiences and satisfaction using these methods in the private sector in Nigeria and the public sector in Zambia.

**Methods:**

We did a prospective cohort study of long-acting reversible contraceptive users across 40 private sector clinics in Nigeria and 21 public sector clinics in Zambia. Eligible women were aged 18–49 years in Nigeria and 16–49 years in Zambia, had chosen to receive the hormonal intrauterine device, copper intrauterine device, or implant (either a 5-year levonorgestrel-releasing subdermal implant or a 3-year etonogestrel-releasing subdermal implant), and, in Nigeria only, had access to a telephone. Women were interviewed within 100 days of receiving their contraceptive method either via telephone in Nigeria or in person in Zambia, with follow-up surveys at 6 months and 12 months. The primary outcomes were method-specific, 12-month continuation rates—ie, continuation rates of the hormonal intrauterine device, copper intrauterine device, and implant across Nigeria and Zambia. We used Kaplan-Meier methods to estimate the cumulative probabilities of method-specific continuation and a log-rank test to compare contraceptive methods. We analysed self-reported satisfaction and experiences as a secondary outcome.

**Findings:**

Between June 25 and Nov 22, 2018, we enrolled a total of 1542 women (n=860 in Nigeria and n=682 in Zambia) receiving a long-acting reversible contraceptive. In total, 835 women (266 [32%] hormonal intrauterine device users, 274 [33%] copper intrauterine device users, and 295 [35%] implant users) in Nigeria and 367 (140 [38%] hormonal intrauterine device users, 149 [40%] copper intrauterine device users, and 78 [21%] implant users) in Zambia were included in the study analysis. The 12-month cumulative continuation rates were 86·8% (95% CI 82·1–90·4) for the hormonal intrauterine device, 86·9% (82·1–90·4) for the copper intrauterine device, and 85·0% (80·2–88·7) for implants in Nigeria. In Zambia, the 12-month cumulative continuation rates were 94·7% (89·2–97·4) for the hormonal intrauterine device, 89·1% (82·3–93·4) for the copper intrauterine device, and 83·1% (72·2–90·1) for implants. At least 71% of respondents across the timepoints were very satisfied with their method, and at least 55 (79%) of 70 reported having recommended their contraceptive method to someone else. Across the methods, the most commonly self-reported positive aspect of long-acting reversible contraceptive use at 12 months was effectiveness in Nigeria (range 93–94%) and long-lasting duration in Zambia (48–60%). Between 124 (50%) of 248 and 136 (59%) of 230 Nigerian participants and 26 (42%) of 62 and 66 (57%) of 117 Zambian participants reported nothing negative about their contraceptive method.

**Interpretation:**

Our study showed high continuation rates and satisfaction across long-acting reversible contraceptives, including the hormonal intrauterine device, a method that has been largely underused in sub-Saharan Africa. This finding supports the inclusion of the hormonal intrauterine device as a valuable addition to the mix of contraceptive methods in Nigeria and Zambia.

**Funding:**

Bill & Melinda Gates Foundation.

## Introduction

Long-acting reversible contraceptives, including hormone-releasing intrauterine devices, copper intrauterine devices, and subdermal implants, are highly efficacious, have few contraindications, and do not require user involvement or resupply visits after insertion.^1^ An analysis of data from the Demographic and Health Surveys in 21 countries indicated that implants and copper intrauterine devices have higher continuation rates than short-acting contraceptive methods.^2^ Recent efforts to expand access to implants have led to substantial increases in their use in a number of low-income and middle-income countries.^3^, ^4^ However, the use of the copper intrauterine device remains low in many of these countries because of barriers related to both supply and demand.^5^

**Research in context**

**Evidence before this study**

We did a search in PubMed for articles published between Jan 1, 1990, and March 31, 2021, with terms commonly used for the levonorgestrel-releasing intrauterine device, with a focus on use in low-income countries. We found evidence showing high satisfaction and continuation rates for the hormonal intrauterine device in developed countries. However, evidence in low-income and middle-income countries is inadequate. Although the contraceptive methods available in sub-Saharan African countries typically include the copper intrauterine device and implants, the hormonal intrauterine device has not been widely available largely because of price barriers. Several introduction programmes have recently supported provision of this contraceptive method, and plans exist to increase availability in the public sector, including in Nigeria and Zambia, as more affordable products become available. Existing evidence among hormonal intrauterine users in low-income and middle-income countries is either limited to special populations (eg, post-partum women) or does not include comparative data for other long-acting reversible contraceptives.

**Added value of this study**

To the best of our knowledge, this study is the first to provide side-to-side satisfaction evidence for all long-acting reversible contraceptive options—inclusive of the hormonal intrauterine device, copper intrauterine device, and implants—among a general population in low-income and middle-income countries over a 12-month timeframe. We found that continuation rates for the hormonal intrauterine device were comparable or favourable to other long-acting reversible contraceptives, and that satisfaction was high across the contraceptive methods. Moreover, the results are consistent across two different settings. Although menstrual disruption is a common cause of discontinuation of or not using contraception in sub-Saharan Africa, our findings add to the body of knowledge contributing a more nuanced understanding of women's experiences of various types of bleeding changes.

**Implications of all the available evidence**

30 years after initial introduction in Europe, several governments are committing to increasing the availability of the hormonal intrauterine device in low-income and middle-income countries, including in Nigeria and Zambia. A key consideration for scale-up is whether the method is acceptable to women in these settings. Our findings add to existing evidence by showing comparable or favourable satisfaction and continuation rates relative to other long-acting reversible contraceptives over a 12-month timeframe in two different settings, suggesting that the hormonal intrauterine device could be an important addition in the context of full contraceptive method choice.

Mirena (Bayer, Turku, Finland), the innovator hormonal intrauterine device product, was first introduced in Europe in 1990 and in the USA in 2000, and this contraceptive method has been popular in these settings. However, the hormonal intrauterine device has not been widely available in low-income and middle-income countries largely because of high commodity prices.^6^ Yet, the global landscape is changing: in 2015, a consortium of donors, governments, manufacturers, researchers, and service delivery groups initiated a collaboration to support expanded access to this contraceptive method.^7^ Now, the US Agency for International Development and the United Nations Population Fund—the two biggest procurement agencies for family planning globally—have added the hormonal intrauterine device to their product catalogues for the first time and several countries, including Nigeria and Zambia, plan to introduce it as part of the contraceptive method mix in the public sector.^8^

With two pregnancies per 1000 women in 1 year of typical use, the level of effectiveness of the hormonal intrauterine device is four times that of the copper intrauterine device, 35 times that of injectables, and 70 times that of pills.^9^, ^10^ The slow and steady localised release of hormones into the uterus results in minimal side-effects that might be more tolerable than those of other hormonal methods.^11^ Moreover, the hormonal intrauterine device has distinctive non-contraceptive properties, including reduced menstrual bleeding; it is a proven treatment for several conditions, including heavy menstrual bleeding; and it has the potential to reduce iron deficiency anaemia.^12^ Additionally, the available evidence shows high continuation rates for the hormonal intrauterine device. For example, a randomised controlled trial involving nine countries reported 1-year continuation rates of 84% for the hormonal intrauterine device and 90% for the copper intrauterine device.^13^ In a prospective cohort study in the USA, the hormonal intrauterine device had a 1-year continuation rate of 87% compared with 84% for the copper intrauterine device and 82% for the 3-year subdermal implant.^14^

Evidence for the use of the hormonal intrauterine device in sub-Saharan Africa remains inadequate, largely because of low availability of this contraceptive method. A cohort study of post-partum women in Kenya found no significant differences between 12-month continuation rates among hormonal intrauterine device users and implant users (89% *vs* 92%). Levels of satisfaction were higher for the hormonal intrauterine device users than for the implant users after 6 months of use (87% *vs* 75%), although the gap narrowed by 12 months (87% *vs* 84%).^15^ An observational study, focusing on the post-partum period, reported that 86% of hormonal intrauterine device users interviewed 3–6 months after insertion in Kenya, and 79% of those interviewed in Zambia with an average time of 10 months after insertion were still using this method.^16^ Qualitative data in Kenya, Nigeria, and Zambia suggest that key opinion leaders, providers, and users recognise the advantages of the hormonal intrauterine device, and support its inclusion in the contraceptive method mix.^17^, ^18^, ^19^ In this study, we therefore aimed to expand the knowledge about the use of the hormonal intrauterine device, copper intrauterine device, and subdermal implant by measuring continuation rates at 6 months and 12 months and assessing women's experiences and satisfaction using these methods in sub-Saharan Africa in the private sector in Nigeria and the public sector in Zambia.

## Methods

### Study design and participants

We did a prospective cohort study of long-acting reversible contraceptive users at sites supported by the Society for Family Health (SFH) in Nigeria and Zambia. Eligible women were aged 18–49 years in Nigeria and 16–49 years in Zambia, had chosen to receive the hormonal intrauterine device, copper intrauterine device, or implant (either a 5-year levonorgestrel-releasing subdermal implant or a 3-year etonogestrel-releasing subdermal implant), and, in Nigeria only, had access to a telephone. In Nigeria, health-care providers from 40 private sector clinics across 18 states referred women to the study between June 25 and Nov 15, 2018. In Zambia, health-care providers referred women from 21 public sector clinics in Copperbelt and Muchinga provinces between July 31 and Nov 22, 2018. We received ethical approval from the National Health Research Ethics Committee in Nigeria; the Excellence in Research Ethics and Science and the National Health Research Authority in Zambia; and FHI 360's Protection of Human Subjects Committee in the USA. We obtained oral consent from participants before all interviews in Nigeria. In Zambia, we obtained written consent from participants at baseline and verbal consent at subsequent interview rounds.

### Procedures

SFH introduced hormonal intrauterine devices donated by the International Contraceptive Access Foundation through private sector clinics in its social franchise network in Nigeria and through SFH-supported public sector clinics in Zambia, both in 2017. All long-acting reversible contraceptives were free to clients in Zambia, whereas SFH recommended Nigerian providers charge a small service fee (3000 Naira [₦] for the hormonal intrauterine device, ₦1500 for implants, and ₦1000 for the copper intrauterine device).

We interviewed eligible participants within 100 days of receiving their contraceptive method, with follow-ups after 6 months and 12 months to collect data on their experiences. We compensated participants ₦1000 at each timepoint in Nigeria and reimbursed 50 Kwacha for any interview requiring travel in Zambia. At each follow-up interview, we asked the women whether they were still using their contraceptive method; and if not, how long they had it before it was removed. We also asked women about satisfaction with their method, positive and negative aspects of method use, side-effects and bleeding changes, and, as applicable, removal intentions or experiences and subsequent contraceptive method use. We developed survey questionnaires based on the existing literature, and adjusted them through peer review, extensive discussions with local study staff, and a test of the survey with women with similar characteristics to those of study participants.

Given practical considerations related to geographical dispersion of study sites and characteristics of the target population, data collection took place by telephone in Nigeria and in person at the health facility, women's homes, or another agreed-upon location in Zambia. Trained research assistants did all interviews in the local languages using electronic tablets. Supervisors independently verified information about the baseline method received and insertion date from clinic records.

### Outcomes

The primary outcomes were method-specific, 12-month continuation rates—ie, continuation rates of the hormonal intrauterine device, copper intrauterine device, and implant in the private sector in Nigeria and the public sector in Zambia. Secondary outcomes were the method-specific, 6-month continuation rates and the women's experiences and satisfaction using these methods at 6 months and 12 months.

### Statistical analysis

We planned to enrol 854 women in Nigeria (276 hormonal intrauterine device users, 276 copper intrauterine device users, and 302 implant users) and 415 women (161 hormonal intrauterine device users, 161 copper intrauterine device users, and 93 implant users) in Zambia to estimate continuation rates with 95% CIs with a 5% precision for each contraceptive method by country. We inflated the sample size to account for potential clustering effects using an intraclass correlation of 3% to be conservative. We used available information about continuation rates of long-acting reversible contraceptives in the two countries (we applied identical assumptions for the hormonal intrauterine device and copper intrauterine device) for these sample size calculcations.^2^ On the basis of experience with other ongoing, longitudinal research, we assumed the loss to follow-up over the course of the study would be 45% on the telephone in Nigeria and 30% in person in Zambia.

We estimated the continuation rates as the proportion of women who reported continuation at 12 months. This analysis, however, uses data for women with complete follow-up information, inclusive of women who reported discontinuation at 6 months and those surveyed at 12 months. Therefore, we also used Kaplan-Meier time-to-event probabilities to estimate method-specific cumulative continuation rates, which use all follow-up time contributed by the participants, including time to discontinuation as well as incomplete follow-up time as censored event time. We used self-reported duration of use at the time of removal to calculate the time to event. We defined self-reported removals as discontinuation events, regardless of whether the woman then took up a similar or different contraceptive method. We censored participants who did not report discontinuation at their last completed survey. Censoring time was estimated as 0 weeks for baseline, 26 weeks for the 6-month survey, and 52 weeks for the 12-month survey.

We did an ex post facto, exploratory comparison of continuation rates across methods with a three-waylog-rank test in each country. If the three-way test was significant, we planned to do additional pairwise comparisons. We did descriptive analyses for all other variables. Because attrition due to early removal introduces bias, the 6-month and 12-month results on satisfaction and experiences were not compared with one another.

We did all the statistical analyses using Stata (version 15).

### Role of the funding source

The funder of the study had no role in study design, data collection, data analysis, data interpretation, or writing of the report.

## Results

**Figure 1:**
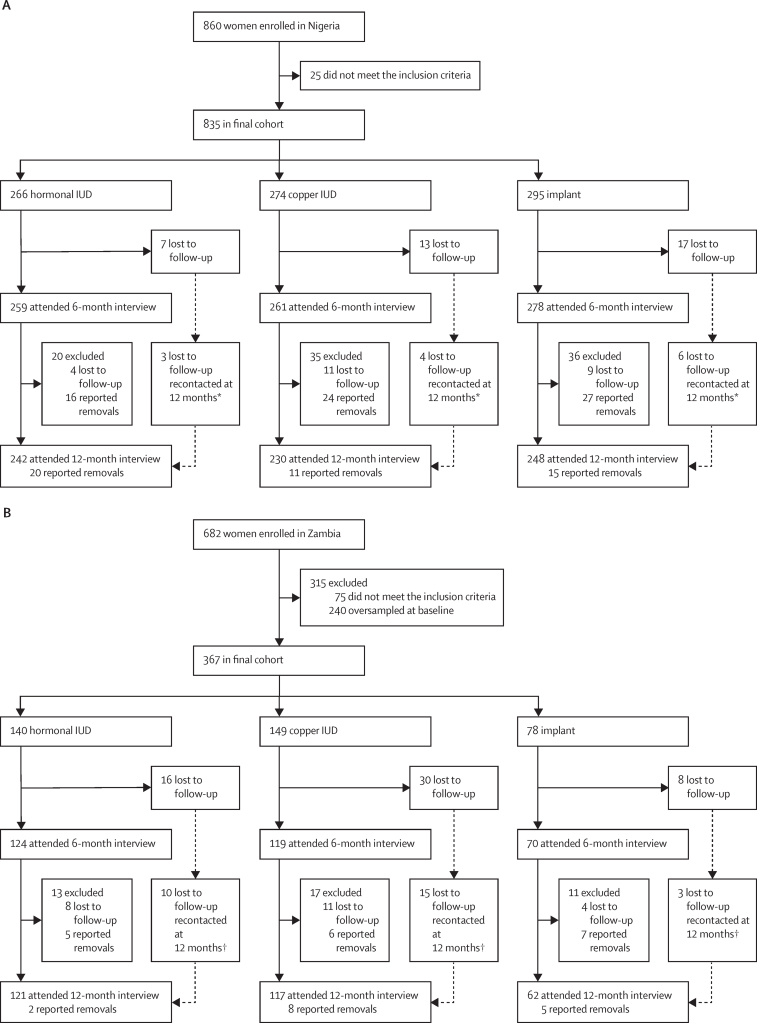
Flow chart for cohort study in Nigeria (A) and Zambia (B) IUD=intrauterine device. *Women not reached at 6 months were recontacted at 12 months, with 13 women completing the 12-month survey and not the 6-month survey. †Women not reached at 6 months were recontacted at 12 months, with 28 women completing the 12-month survey and not the 6-month survey.

Between June 25 and Nov 22, 2018, we enrolled a total of 1542 women (n=860 in Nigeria and n=682 in Zambia) receiving a long-acting reversible contraceptive (figure 1). We excluded 25 women in Nigeria and 75 women in Zambia who did not meet the inclusion criteria for the insertion date upon verification of clinic records. In Zambia, women were oversampled at baseline because of delays in tallying the number of completed surveys due to connectivity issues. We therefore retained women in the Zambian cohort based on the order in which they were enrolled, resulting in 240 long-acting reversible contraceptive users in excess of the planned sample size being excluded from both the 6-month and 12-month follow-ups. In total, the final cohorts consisted of 835 women (266 [32%] hormonal intrauterine device users, 274 [33%] copper intrauterine device users, and 295 [35%] implant users) in Nigeria and 367 (140 [38%] hormonal intrauterine device users, 149 [40%] copper intrauterine device users, and 78 [21%] implant users) in Zambia.

Within each country, the characteristics of the participants were similar across the types of long-acting reversible contraceptives; however, on average, implant users in Zambia were younger and had less years of schooling, and fewer were in the upper wealth quintile (table 1).

**Table 1:** Participant characteristics at baseline

		**Nigeria**			**Zambia**		
		Hormonal IUD (n=266)	Copper IUD (n=274)	Implant (n=295)	Hormonal IUD (n=140)	Copper IUD (n=149)	Implant (n=78)
Age (years)							
	16–17	NA	NA	NA	1 (1%)	0	2 (3%)
	18–24	21 (8%)	12 (4%)	40 (14%)	26 (19%)	15 (10%)	28 (36%)
	25–34	127 (48%)	130 (47%)	148 (50%)	71 (51%)	84 (56%)	38 (49%)
	35–49	118 (44%)	132 (48%)	107 (36%)	42 (30%)	50 (34%)	10 (13%)
	Mean	33·3 (6·1)	33·7 (5·7)	31·7 (6·3)	30·5 (6·4)	32·2 (6·0)	27·1 (6·2)
	Median	34 (29–38)	34 (30–38)	32 (27–36)	30 (26–35)	32 (27–37)	26 (23–31)
Married		258 (97%)	263 (96%)	273 (93%)	105 (75%)	130 (87%)	54 (69%)
Parity							
	0	5 (2%)	2 (1%)	11 (4%)	9 (6%)	4 (3%)	3 (4%)
	1–2	74 (28%)	81 (30%)	104 (35%)	45 (32%)	41 (28%)	43 (55%)
	3–4	137 (52%)	121 (44%)	135 (46%)	60 (43%)	68 (46%)	24 (31%)
	≥5	50 (19%)	70 (26%)	44 (15%)	25 (18%)	36 (24%)	8 (10%)
	Mean number of children	3·3 (1·5)	3·5 (1·7)	3·1 (1·7)	3·1 (1·9)	3·4 (1·7)	2·4 (1·6)
	Median number of children	3 (2–4)	3 (2–5)	3 (2–4)	3 (2–4)	3 (2–4)	2 (1–3)
Highest education completed							
	No schooling or some primary	5 (2%)	10 (4%)	11 (4%)	18 (13%)	18 (12%)	13 (17%)
	Primary	31 (12%)	29 (11%)	36 (12%)	50 (36%)	58 (39%)	42 (54%)
	Secondary	113 (42%)	127 (46%)	136 (46%)	55 (39%)	59 (40%)	23 (29%)
	More than secondary	117 (44%)	108 (39%)	112 (38%)	17 (12%)	14 (9%)	0
Urban wealth quintile*							
	Lowest	4 (2%)	5 (2%)	7 (2%)	19 (14%)	15 (10%)	7 (9%)
	Second	23 (9%)	16 (6%)	22 (7%)	12 (9%)	11 (7%)	10 (13%)
	Middle	29 (11%)	31 (11%)	38 (13%)	27 (19%)	26 (18%)	29 (38%)
	Fourth	50 (19%)	54 (20%)	67 (23%)	37 (26%)	38 (26%)	22 (29%)
	Highest	160 (60%)	166 (61%)	159 (54%)	45 (32%)	58 (39%)	9 (12%)

Data are n (%), mean (SD), or median (IQR). Because of small amounts of missing data, not all data sum to the totals in the table headings. IUD=intrauterine device. NA=not applicable.

* Relative wealth was measured using the Equity Tool.^20^ The urban version of the Equity Tool compares participants to the urban population.

As a validity check, we compared self-reported durations of use against the time elapsed between insertion dates obtained from clinic records and interview dates. In Nigeria, we censored the time of one woman in the analysis at 6 months and two women at 12 months because of invalid reported durations of use, but with reported durations higher than possible according to the visit date. All observations in Zambia passed the validity check.

The intial analysis uses data from participants who completed the 12-month follow-up and those who reported discontinuation at 6 months (n=787 in Nigeria and n=318 in Zambia). In Nigeria, the proportions of women reporting continuation of their long-acting reversible contraceptive at 12 months were 86·0% (95% CI 81·8–90·3) for the hormonal intrauterine device, 86·2% (82·0–90·5) for the copper intrauterine device, and 84·7% (80·4–89·0) for implants. In Zambia, the proportions of women reporting continuation of their long-acting reversible contraceptive at 12 months were 94·4% (95% CI 90·4–98·5) for the hormonal intrauterine device, 88·6% (82·9–94·3) for the copper intrauterine device, and 82·6% (73·4–91·8) for implants.

**Figure 2:**
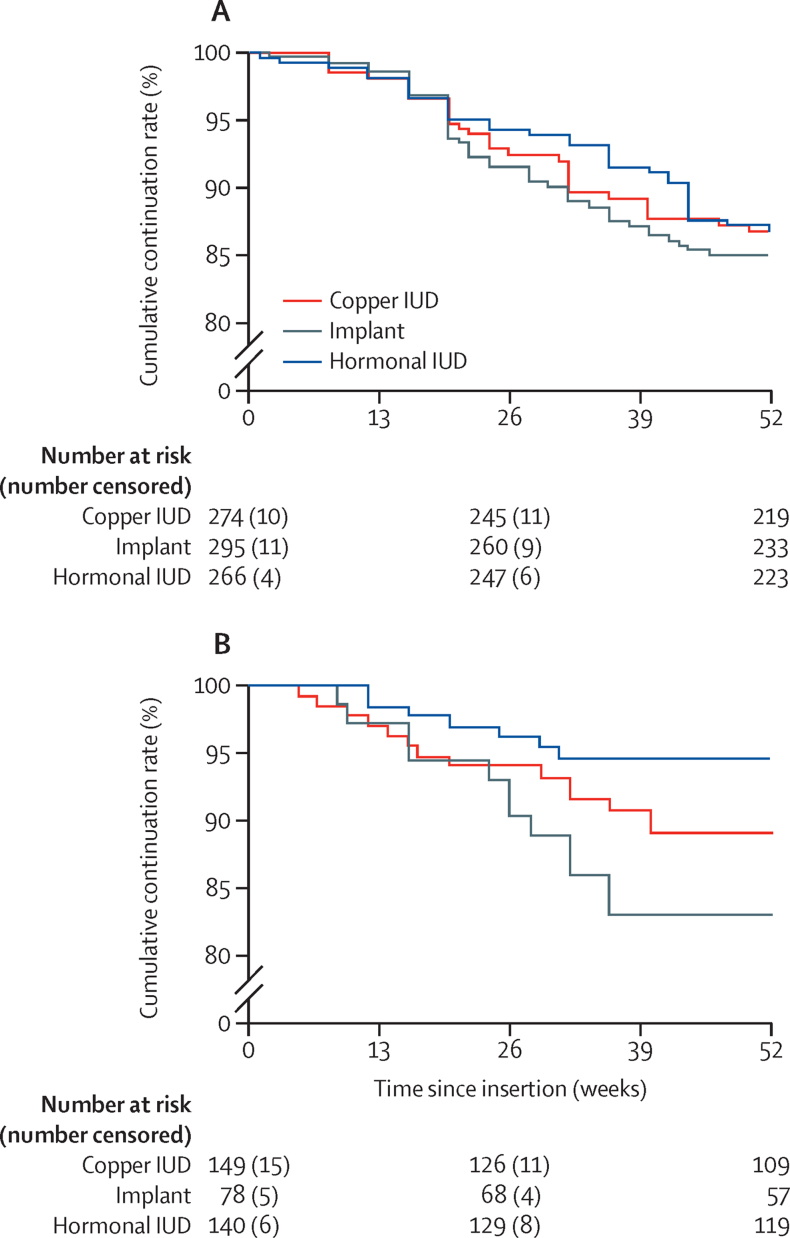
Kaplan-Meier time-to-event curves of the hormonal IUD, copper IUD, and implants in Nigeria (A) and Zambia (B) IUD=intrauterine device.

At 6 months, the cumulative continuation rates based on time-to-event techniques were 94·3% (95% CI 90·7–96·5) in Nigeria and 96·3% (91·3–98·4) in Zambia for the hormonal intrauterine device, 92·4% (88·5–95·0) in Nigeria and 94·0% (88·4–97·0) in Zambia for the copper intrauterine device, and 91·6% (87·7–94·3) in Nigeria and 90·4% (80·9–95·3) in Zambia for implants. At 12 months, the cumulative continuation rates were 86·8% (82·1–90·4) in Nigeria and 94·7% (89·2–97·4) in Zambia for the hormonal intrauterine device, 86·9% (82·1–90·4) in Nigeria and 89·1% (82·2–93·4) in Zambia for the copper intrauterine device, and 85·0% (80·2–88·7) in Nigeria and 83·1% (72·2–90·1) in Zambia for implants (table 2; figure 2). No significant differences were found in the Kaplan-Meier time-to-event curves across the three contraceptive methods in Nigeria (p=0·74). In Zambia, the Kaplan-Meier time-to-event curves were significantly different (p=0·033) and pairwise comparisons showed higher continuation among hormonal intrauterine device users than among implant users (p=0·0080), but no significant differences between the copper intrauterine device and the hormonal intrauterine device (p=0·11).

**Table 2:** Cumulative continuation rates and information about removal intentions and status by contraceptive method

		**Nigeria**			**Zambia**		
		Hormonal IUD (n=266)	Copper IUD (n=274)	Implant (n=295)	Hormonal IUD (n=140)	Copper IUD (n=149)	Implant (n=78)
6-month cumulative continuation rate (95% CI)		94·3 (90·7–96·5)	92·4 (88·5–95·0)	91·6 (87·7–94·3)	96·3 (91·3–98·4)	94·0 (88·4–97·0)	90·4 (80·9–95·3)
12-month cumulative continuation rate (95% CI)		86·8 (82·1–90·4)	86·9 (82·1–90·4)	85·0 (80·2–88·7)	94·7 (89·2–97·4)	89·1 (82·3–93·4)	83·1 (72·2–90·1)
Removal intentions and status at 12 months							
	Never wanted to remove	198/258 (77%)	191/254 (75%)	190/275 (69%)	108/126 (86%)	95/123 (77%)	48/69 (70%)
	Wanted removal but never tried	13/258 (5%)	20/254 (8%)	30/275 (11%)	5/126 (4%)	4/123 (3%)	4/69 (6%)
	Wanted removal, tried, but still has method	11/258 (4%)	8/254 (3%)	13/275 (5%)	6/126 (5%)	10/123 (8%)	5/69 (7%)
	Removed	36/258 (14%)	35/254 (14%)	42/275 (15%)	7/126 (6%)	14/123 (11%)	12/69 (17%)
Mean reported duration of use at time of removal (weeks)*		29·3 (14·2)	25·4 (11·4)	25·6 (10·5)	20·7 (7·8)	22·1 (12·3)	24·3 (9·5)
Reasons for removals†							
	Desired pregnancy	10/36 (28%)	12/35 (34%)	7/42 (17%)	1/7 (14%)	1/14 (7%)	0/12
	Increased bleeding	6/36 (17%)	9/35 (26%)	15/42 (36%)	1/7 (14%)	5/14 (36%)	6/12 (50%)
	Bleeding disturbances	4/36 (11%)	1/35 (3%)	4/42 (10%)	3/7 (43%)	1/14 (7%)	3/12 (25%)
	Partner disapproved	4/36 (11%)	0/35	2/42 (5%)	2/7 (29%)	2/14 (14%)	0/12
	Pain or discomfort	1/36 (3%)	2/35 (6%)	6/42 (14%)	0/7	2/14 (14%)	0/12
	Other	12/36 (33%)	10/35 (29%)	8/42 (19%)	1/7 (14%)	4/14 (29%)	3/12 (25%)

Data are n/N (%) or mean (SD), unless otherwise specified. IUD=intrauterine device.

* Among women who reported removal of their contraceptive method.

† Among women who reported removal of their contraceptive method, with multiple responses possible.

579 (74%) of 787 women in Nigeria and 251 (79%) of 318 in Zambia reported that they had never considered removing their contraceptive method during the first year (table 2). 113 (14%) of 787 women in Nigeria and 33 (10%) of 318 in Zambia reported having their method removed. The main reasons for removal in Nigeria were increased bleeding with method use and desired pregnancy, whereas in Zambia, the main reasons were bleeding disturbances (ie, irregular periods or spotting) for the hormonal intrauterine device and increased bleeding for the copper intrauterine device and implants.

Women reported similar experiences with their contraceptive method at 6 months and 12 months (appendix; table 3). In Nigeria, 224 (87%) of 259 women using the hormonal intrauterine device in the 6-month sample were very satisfied with their method compared with 197 (71%) of 278 using an implant and 204 (78%) of 261 using the copper intrauterine device. In the 12-month sample, 218 (90%) of 242 hormonal intrauterine device users and 211 (92%) of 230 copper intrauterine device users reported being very satisfied. High satisfaction was less common in implant users, with 207 (83%) of 248 users reporting being very satisfied. In Zambia, the 6-month results show a slightly higher proportion of very satisfied users among the copper intrauterine device (98 [82%] of 119) compared with the hormonal intrauterine device (97 [78%] of 124) and implant (53 [76%] of 70), but this proportion was at least 9% greater among hormonal intrauterine device users (101 [83%] of 121) compared with the copper intrauterine device (86 [74%] of 117) and implant (45 [73%] of 62) in the 12-month sample. At least 55 (79%) of 70 women across the timepoints, contraceptive methods, and countries reported they had recommended their method to someone else.

**Table 3:** Self-reported satisfaction and experiences with contraceptive methods after 12 months of use

		**Nigeria**			**Zambia**		
		Hormonal IUD (n=242)	Copper IUD (n=230)	Implant (n=248)	Hormonal IUD (n=121)	Copper IUD (n=117)	Implant (n=62)
Satisfied with method							
	Very satisfied	218 (90%)	211 (92%)	207 (83%)	101 (83%)	86 (74%)	45 (73%)
	Somewhat satisfied	20 (8%)	18 (8%)	30 (12%)	17 (14%)	28 (24%)	11 (18%)
	Neither satisfied nor dissatisfied	2 (1%)	1 (<1%)	9 (4%)	2 (2%)	1 (1%)	5 (8%)
	Somewhat dissatisfied	1 (<1%)	0	0	0	1 (1%)	0
	Very dissatisfied	1 (<1%)	0	2 (1%)	1 (1%)	1 (1%)	1 (2%)
	Recommended method to someone else	226 (93%)	219 (95%)	233 (94%)	98 (82%)	94 (82%)	53 (85%)
Satisfied with bleeding pattern							
	Very happy	161 (67%)	135 (59%)	129 (52%)	73 (60%)	75 (64%)	34 (55%)
	Somewhat happy	52 (21%)	79 (34%)	80 (32%)	36 (30%)	34 (29%)	16 (26%)
	Neither happy nor unhappy	23 (10%)	14 (6%)	31 (13%)	7 (6%)	4 (3%)	6 (10%)
	Somewhat unhappy	4 (2%)	1 (<1%)	6 (2%)	2 (2%)	1 (1%)	1 (2%)
	Very unhappy	2 (1%)	1 (<1%)	2 (1%)	3 (2%)	3 (3%)	5 (8%)
Self-reported positive aspects of method use*							
	Effective	228 (94%)	216 (94%)	231 (93%)	25 (21%)	16 (14%)	10 (16%)
	Convenient	134 (55%)	126 (55%)	135 (54%)	50 (41%)	54 (46%)	20 (32%)
	Regular periods	43 (18%)	48 (21%)	36 (15%)	13 (11%)	30 (26%)	11 (18%)
	Few side-effects	96 (40%)	96 (42%)	95 (38%)	19 (16%)	13 (11%)	2 (3%)
	Reduced or no bleeding	52 (21%)	3 (1%)	11 (4%)	37 (31%)	5 (4%)	11 (18%)
	Long lasting	33 (14%)	88 (38%)	28 (11%)	72 (60%)	69 (59%)	30 (48%)
	Treats heavy or painful period	28 (12%)	47 (20%)	35 (14%)	29 (24%)	0	1 (2%)
	Discreet	28 (12%)	47 (20%)	35 (14%)	32 (26%)	38 (32%)	13 (21%)
Self-reported negative aspects of method use*							
	Bleeding disturbances†	40 (17%)	11 (5%)	34 (14%)	7 (6%)	19 (16%)	13 (21%)
	More bleeding	11 (5%)	35 (15%)	39 (16%)	5 (4%)	9 (8%)	5 (8%)
	No period	14 (6%)	2 (1%)	18 (7%)	17 (14%)	4 (3%)	9 (15%)
	Insertion painful	2 (1%)	2 (1%)	1 (<1%)	8 (7%)	11 (9%)	1 (2%)
	Device lasts too long	1 (<1%)	1 (<1%)	4 (2%)	14 (12%)	9 (8%)	5 (8%)
	Nothing negative	129 (53%)	136 (59%)	124 (50%)	60 (50%)	66 (56%)	26 (42%)
Self-reported bleeding changes*							
	Lighter period	75 (31%)	8 (3%)	10 (4%)	43 (36%)	10 (9%)	3 (5%)
	Shorter period	46 (19%)	5 (2%)	8 (3%)	52 (43%)	14 (12%)	12 (19%)
	Period stopped	56 (23%)	3 (1%)	67 (27%)	19 (16%)	5 (4%)	13 (21%)
	Bleeding disturbances†	77 (32%)	33 (14%)	57 (23%)	13 (11%)	22 (19%)	14 (23%)
	Heavier period	11 (5%)	90 (39%)	33 (13%)	8 (7%)	18 (15%)	6 (10%)
	Longer period	12 (5%)	24 (10%)	74 (30%)	6 (5%)	11 (9%)	8 (13%)
	No change	78 (32%)	110 (48%)	86 (35%)	40 (33%)	61 (52%)	17 (27%)
Reported any side-effects		81 (33%)	66 (29%)	79 (32%)	22 (18%)	18 (15%)	15 (24%)
Reported a problem about method to provider		99 (41%)	98 (43%)	101 (41%)	38 (31%)	38 (34%)	18 (29%)
Self-reported effect of reduced bleeding on life‡							
	Positive effect	72/132 (55%)	2/15 (13%)	37/79 (47%)	50/64 (78%)	16/22 (73%)	19/26 (73%)
	No effect	54/132 (41%)	13/15 (87%)	40/79 (51%)	12/64 (19%)	5/22 (23%)	2/26 (8%)
	Negative effect	6/132 (5%)	0	2/79 (3%)	2/64 (3%)	1/22 (5%)	5/26 (19%)
Self-reported change in amounts of menstrual products used§							
	More products	6/185 (3%)	40 (19%)	40/198 (20%)	9/112 (8%)	21/105 (20%)	8/53 (15%)
	Same amount	95/185 (51%)	163/216 (75%)	131/198 (66%)	18/112 (16%)	45/105 (43%)	15/53 (28%)
	Fewer products	81/185 (44%)	8/216 (4%)	20/198 (10%)	77/112 (69%)	38/105 (36%)	30/53 (57%)
	Different products	3/185 (2%)	5/216 (2%)	7/198 (4%)	8/112 (7%)	1/105 (1%)	0

Data are n (%) or n/N (%). Due to small amounts of missing data, not all data sum to the totals in the table headings. IUD=intrauterine device.

* Multiple responses possible.

† Bleeding disturbances include irregular periods or spotting.

‡ Among women who reported a lighter period, shorter period, or no period.

§ Among women still using the contraceptive method at 12 months compared with before receiving the method.

More women reported experiencing changes in their menses than other side-effects. Self-reported patterns of bleeding changes varied; however, results on the most common bleeding patterns were generally consistent across timepoints and between the two countries. In both countries, at 12 months, more women using the hormonal intrauterine device reported lighter (75 [31%] of 242 in Nigeria and 43 [36%] of 121 in Zambia) and shorter periods (46 [19%] in Nigeria and 52 [43%] in Zambia) compared with users of the copper intrauterine device and implant (table 3). In Nigeria, 77 (32%) of 242 women using the hormonal intrauterine device reported bleeding disturbances, and 56 (23%) of 242 reported amenorrhoea (table 3). For the copper intrauterine device, 110 (48%) of 230 women in Nigeria and 61 (52%) of 117 women in Zambia reported no change in their period. The predominant self-reported bleeding change in Nigeria for the copper intrauterine device was heavier menstrual bleeding (90 [39%] of 230), whereas in Zambia, 18 (15%) of 117 women reported heavier menstrual bleeding and 22 (19%) reported bleeding disturbances. For the implant, bleeding disturbances were reported in 57 (23%) of 248 women in Nigeria and 14 (23%) of 62 in Zambia, amenorrhoea in 67 (27%) in Nigeria and 13 (21%) in Zambia, and a longer period in 74 (30%) in Nigeria and eight (13%) in Zambia. Overall, hormonal intrauterine device users and copper intrauterine device users were most satisfied with their bleeding pattern; satisfaction levels were consistently the lowest among implant users.

Women reported similar positive aspects of method use at 12 months across the three long-acting reversible contraceptives. In Nigeria, these included effectiveness (range 93–94%), convenience (54–55%), and few side-effects (38–42%), whereas in Zambia they were long lasting (48–60%), convenience (32–46%), and discreet use (21–32%). 52 (21%) of 242 women using the hormonal intrauterine device in Nigeria and 37 (31%) of 121 in Zambia identified reduced or no bleeding as a benefit; 28 (12%) of 242 in Nigeria and 29 (24%) of 121 in Zambia saw this contraceptive method as a potential treatment for heavy or painful periods as an advantage (table 3). Across the methods, between 124 (50%) of 248 and 136 (59%) of 230 Nigerian participants and 26 (42%) of 62 and 66 (56%) of 117 Zambian participants reported experiencing nothing negative about their contraceptive method.

Among women who reported having a lighter period, shorter period, or amenorrhoea across the contraceptive methods, between 13 (45%) of 29 and 26 (87%) of 30 women at 6 months and between 16 (73%) of 22 and 50 (78%) of 64 women at 12 months in Zambia reported reduced or no bleeding as having a positive effect on their lives (table 3, appendix). In Nigeria, 90 (51%) of 177 hormonal intrauterine device users at 6 months and 72 (55%) of 132 at 12 months reported a positive effect from reduced or no bleeding, whereas 36 (63%) of 57 copper intrauterine device users at 6 months and 13 (87%) of 15 at 12 months, as well as 81 (64%) of 127 implant users at 6 months and 40 (51%) of 79 at 12 months indicated that reduced or no bleeding had had no effect on their lives. Apart from the 6-month sample in Zambia, the proportion of women rating the effect of reduced or no bleeding on their lives as positive was consistently highest in the hormonal intrauterine device group (appendix; table 3).

Additionally, more women using the hormonal intrauterine device reported a reduction in the quantity of menstrual products used to manage their period compared with before they received their contraceptive method relative to those using the copper intrauterine device or implant. For example, in the 6-month sample, 128 (59%) of 218 women using the hormonal intrauterine device in Nigeria and 84 (72%) of 116 in Zambia reported a reduction in the amount of menstrual products used compared with 32 (14%) of 230 using the copper intrauterine device and 58 (27%) of 217 using an implant in Nigeria and 47 (43%) of 110 using the copper intrauterine device and 30 (54%) of 56 using an implant in Zambia (appendix).

## Discussion

Findings from two different settings show high continuation rates and satisfaction across long-acting reversible contraceptives, including the hormonal intrauterine device, a method that has been largely underused in sub-Saharan Africa. Although preferences were not uniform, all long-acting reversible contraceptives shared characteristics that women appreciated within each country, including high effectiveness, convenience, and few side-effects in Nigeria and duration of protection, convenience, and discreetness in Zambia. Reduced or no bleeding emerged as an additional distinctive benefit of the hormonal intrauterine device in both countries.

Overall, although women's experiences varied, patterns of contraceptive-induced bleeding changes were consistent in our study with what has been reported for each long-acting reversible contraceptive in other studies.^21^, ^22^, ^23^ In general, side-effects are a key cause of discontinuation in sub-Saharan Africa.^24^ Although the Demographic and Health Surveys did not until recently differentiate between bleeding changes and non-bleeding side-effects, other available evidence suggests that bleeding changes can be particularly problematic.^25^ In our study, more women reported bleeding changes than other side-effects. Overall, bleeding disturbances inclusive of irregular periods and spotting, and increased bleeding emerged as negative aspects of using some long-acting reversible contraceptives. Although small sample sizes limit our ability to draw strong conclusions, increased bleeding and bleeding disturbances were also reasons for removing or wanting to remove a contraceptive method. At the same time, our findings highlight that reduced bleeding or amenorrhoea can have a positive effect on women's lives, which is consistent with other recent data from Nigeria and Zambia.^17^, ^26^ Also, a large proportion of women using the hormonal intrauterine device reported using fewer menstrual products compared with before starting their method relative to other long-acting reversible contraceptives. Documented concerns associated with the financial burden of menstrual hygiene management suggest this effect could be an important benefit.^27^, ^28^ In settings where concerns about amenorrhoea tend to persist, this effect could have the potential to increase acceptability.^29^

The prospective cohort study design documenting the experiences of women using long-acting reversible contraceptives in real-world conditions and the use of time-to-event methods to account for attrition data confer some strength to this study. However, some limitations must be acknowledged. Sample sizes were based on estimating method-specific continuation rates and do not support comparisons between contraceptive methods. Results from the log-rank test comparing the continuation rates across methods must be interpreted cautiously because of their ad-hoc nature and the potentially low statistical power. The study population is also limited to the settings in which the method was introduced in each country, and to women with telephones in Nigeria. As such, our results might not be representative of the broader population of clinic clients, as the study does not use probability sampling based on a comprehensive sampling frame. The possibility of selection bias exists, as the recruitment approach might have missed users of long-acting reversible contraceptives who were not referred by providers or otherwise not available for the study. However, because recruitment occurred at uptake, this bias is not based on experiences with the method and therefore should not affect our key outcomes. In addition, the users of long-acting reversible contraceptives selected their own method, which is a strength of these data for the estimation of continuation rates in real-world conditions, but it means that method users cannot be directly compared. Furthermore, attrition can bias our results if loss to follow-up is informative. Fortunately, we did not have much attrition in our cohorts. Reliance on self-reports carries a risk of reporting bias and of missing expulsions that might have gone unnoticed by users and limits our ability to rigorously document side-effects and bleeding changes. Although the amount of the compensation was chosen carefully, courtesy bias also cannot be entirely ruled out. Whereas we used clinic records to confirm method received and insertion dates, independently verifying client reports on status of the contraceptive method used was not feasible. Although most women using the hormonal intrauterine device were new to their method, we did not require those using the copper intrauterine device or implant to be first-time users; as such, findings comparing the hormonal intrauterine device to other methods might be conservative since users of other long-acting reversible contraceptives might have included repeat, satisfied users. Finally, we did not differentiate between types of implants.

In conclusion, our results are timely for Nigeria and Zambia, given current efforts to introduce the hormonal intrauterine device at a large scale.^30^ The findings showing high continuation rates and satisfaction are encouraging. The comparable or favourable results of the hormonal intrauterine device relative to implants provide an important acceptability benchmark given the popularity of implants in sub-Saharan Africa. In addition, non-contraceptive benefits of the hormonal intrauterine device, including reduced bleeding and potential for fewer side-effects, might make it a particularly attractive option for some women and should be emphasised in efforts to generate demand. In the context of contraceptive method choice, the hormonal intrauterine device can be a valuable addition to increase the range of contraceptive methods available in sub-Saharan Africa.

## Data sharing

De-identified datasets are publicly available online through the Harvard Dataverse.

## Declaration of interests

We declare no competing interests.
