# Supplementary material for: Expanding long-acting contraceptive options: a prospective cohort study of the hormonal intrauterine device, copper intrauterine device, and implants in Nigeria and Zambia
Source: Lancet Glob Health. 2021 Aug 30;9(10):e1431–41. doi: 10.1016/S2214-109X(21)00318-1 (PMC8440225; doi:10.1016/S2214-109X(21)00318-1)
Supplement: Supplementary appendix [file mmc1.pdf]

THE LANCET
Global Health

Supplementary appendix

This appendix formed part of the original submission and has been peer reviewed. We post it as supplied by the authors.

Supplement to: Brunie A, Stankevitz K, Nwala AA, et al. Expanding long-acting contraceptive options: a prospective cohort study of the hormonal intrauterine device, copper intrauterine device, and implants in Nigeria and Zambia. *Lancet Glob Health* 2021; published online Aug 30. http://dx.doi.org/10.1016/S2214-109X(21)00318-1.

Supplementary appendix

Appendix table 1. Self-reported satisfaction and experiences with methods in the 6-month sample by method

	Nigeria			Zambia		
	**Hormonal IUD**	**Copper IUD**	**Implant**	**Hormonal IUD**	**Copper IUD**	**Implant**
	**n=259**	**n=261**	**n=278**	**n=124**	**n=119**	**n=70**
**Satisfied with method**						
Very satisfied	224 (86·5)	204 (78·2)	197 (70·9)	97 (78·2)	98 (82·4)	53 (75·7)
Somewhat satisfied	27 (10·4)	42 (16·1)	61 (21·9)	13 (10·5)	18 (15·1)	15 (21·4)
Neither satisfied nor dissatisfied	6 (2·3)	9 (3·5)	12 (4·3)	8 (6·5)	1 (0·8)	2 (2·9)
Somewhat dissatisfied	1 (0·4)	4 (1·5)	3 (1·1)	1 (0·8)	1 (0·8)	0 (0·0)
Very dissatisfied	1 (0·4)	2 (0·8)	5 (1·8)	5 (4·0)	1 (0·8)	0 (0·0)
**Recommended method to someone else**	230 (88·8)	225 (86·2)	225 (80·9)	109 (87·9)	97 (81·5)	55 (78·6)
**Satisfied with bleeding pattern**						
Very happy	186 (71·8)	155 (59·6)	128 (46·0)	79 (63·7)	82 (68·9)	38 (54·3)
Somewhat happy	47 (18·2)	56 (21·5)	75 (27·0)	29 (23·4)	29 (24·4)	21 (30·0)
Neither happy nor unhappy	21 (8·1)	32 (12·3)	50 (18·0)	8 (6·5)	2 (1·7)	5 (7·1)
Somewhat unhappy	3 (1·2)	12 (4·6)	9 (3·2)	2 (1·6)	2 (1·7)	3 (4·3)
Very unhappy	2 (0·8)	5 (1·9)	16 (5·8)	6 (4·8)	4 (3·4)	3 (4·3)
**Self-reported bleeding changes** [a]						
Lighter period	123 (47·5)	34 (13·1)	35 (12·6)	24 (19·4)	10 (8·4)	3 (4·3)
Shorter period	114 (44·0)	39 (15·0)	36 (13·0)	30 (24·2)	18 (15·1)	9 (12·9)
Period stopped	53 (20·5)	10 (3·9)	81 (29·1)	15 (12·1)	8 (6·7)	17 (24·3)
Bleeding disturbances	76 (29·3)	44 (16·9)	97 (34·9)	19 (15·3)	15 (12·6)	18 (25·7)
Heavier period	10 (3·9)	102 (39·2)	47 (16·9)	12 (9·7)	18 (15·1)	7 (10·0)
Longer period	16 (6·2)	60 (23·0)	68 (24·5)	10 (8·1)	10 (8·4)	10 (14·3)
No change	44 (17·0)	91 (34·9)	64 (23·0)	50 (40·3)	63 (52·9)	15 (21·4)
**Reported any side effects**	94 (36·4)	82 (31·4)	111 (39·9)	31 (25·0)	25 (21·0)	16 (22·9)
**Reported a problem about method to provider**	109 (42·3)	106 (40·8)	135 (48·7)	39 (31·5)	31 (26·1)	20 (28·6)
	n=177	n=57	n=127	n=50	n=30	n=29
**Self-reported impact of reduced bleeding on life** [b]						
Positive impact	90 (50·9)	18 (31·6)	38 (29·9)	34 (68·0)	26 (86·7)	13 (44·8)
No impact	81 (45·8)	36 (63·2)	81 (63·8)	11 (22·0)	4 (13·3)	6 (20·7)
Negative impact	6 (3·4)	3 (5·3)	8 (6·3)	5 (10·0)	0 (0·0)	10 (34·5)
	n=218	n=230	n=217	n=116	n=110	n=56
**Self-reported change in amount of menstrual products used** [c d]						
More products	13 (6·0)	70 (30·4)	57 (26·3)	13 (11·2)	18 (16·4)	13 (23·2)
Same amount	75 (34·4)	123 (53·5)	94 (43·3)	14 (12·1)	43 (39·1)	12 (21·4)
Fewer products	128 (58·7)	32 (13·9)	58 (26·7)	84 (72·4)	47 (42·7)	30 (53·6)
Different products	2 (0·9)	5 (2·2)	8 (3·7)	5 (4·3)	2 (1·8)	1 (1·8)

Data are n (%). Due to small amounts of missing data, not all data sum to the totals in the table headings.
[a] Multiple responses possible
[b] Among women who reported experiencing a lighter period, shorter period and/or no period
[c] Among women still using the product at 6 months
[d] Compared to before receiving the method
